# IOTASDN: IOTA 2.0 Smart Contracts for Securing Software-Defined Networking Ecosystem

**DOI:** 10.3390/s24175716

**Published:** 2024-09-02

**Authors:** Mohamed Fartitchou, Ismail Lamaakal, Yassine Maleh, Khalid El Makkaoui, Zakaria El Allali, Paweł Pławiak, Fahad Alblehai, Ahmed A. Abd El-Latif

**Affiliations:** 1 Multidisciplinary Faculty of Nador, Mohammed Premier University, Oujda 60000, Morocco; fartitchou.mohamed@ump.ac.ma (M.F.); ismail.lamaakal@ump.ac.ma (I.L.); k.elmakkaoui@ump.ac.ma (K.E.M.); z.elallali@ump.ac.ma (Z.E.A.); 2 National School of Applied Sciences, Sultan Moulay Slimane University, Beni Mellal 23000, Morocco; 3 Department of Computer Science, Faculty of Computer Science and Telecommunications, Cracow University of Technology, Warszawska 24, 31-155 Krakow, Poland; pawel.plawiak@pk.edu.pl; 4 Institute of Theoretical and Applied Informatics, Polish Academy of Sciences, Bałtycka 5, 44-100 Gliwice, Poland; 5 Computer Science Department, Community College, King Saud University, Riyadh 11437, Saudi Arabia; falblehi@ksu.edu.sa; 6 Jadara University Research Center, Jadara University, Irbid 21110, Jordan; 7 Department of Mathematics and Computer Science, Faculty of Science, Menoufia University, Shebin El-Koom 32511, Egypt

**Keywords:** Blockchain (BC), integrity, IOTA 2.0, security, smart contracts, software-defined networking, trust

## Abstract

Software-Defined Networking (SDN) has revolutionized network management by providing unprecedented flexibility, control, and efficiency. However, its centralized architecture introduces critical security vulnerabilities. This paper introduces a novel approach to securing SDN environments using IOTA 2.0 smart contracts. The proposed system utilizes the IOTA Tangle, a directed acyclic graph (DAG) structure, to improve scalability and efficiency while eliminating transaction fees and reducing energy consumption. We introduce three smart contracts: Authority, Access Control, and DoS Detector, to ensure trusted and secure network operations, prevent unauthorized access, maintain the integrity of control data, and mitigate denial-of-service attacks. Through comprehensive simulations using Mininet and the ShimmerEVM IOTA Test Network, we demonstrate the efficacy of our approach in enhancing SDN security. Our findings highlight the potential of IOTA 2.0 smart contracts to provide a robust, decentralized solution for securing SDN environments, paving the way for the further integration of blockchain technologies in network management.

## 1. Introduction

SDN is driving transformative changes in network management and operations by decoupling the control plane from the data plane. This approach introduces new levels of flexibility, control, and efficiency that were previously unattainable in the rapidly evolving digital landscape. This paradigm shift in networking not only redefines the traditional network architecture but also aligns seamlessly with the dynamic requirements of contemporary digital ecosystems [1,2]. While SDN provides significant advantages in network management and efficiency, it also introduces new security challenges that are essential to address. The centralized architecture of SDN controllers poses a potential single point of failure, rendering the network vulnerable to targeted attacks that could compromise the entire infrastructure.

The dynamic and programmable nature of SDN, while contributing to enhanced network flexibility, also introduces heightened vulnerability to potential attacks from malicious actors. They can use flaws in the software layers to launch attacks like DoS, man-in-the-middle, and data theft. These challenges necessitate robust security mechanisms and policies to ensure the integrity, confidentiality, and availability of network resources in an SDN environment [3,4].

In tackling the security challenges inherent in SDN, machine learning and BC emerge as pivotal solutions [5,6,7]. ML algorithms aim to enhance the SDN network’s capability to intelligently detect, predict, and respond to cyber threats. By analyzing network data, identifying patterns, and learning from past incidents, ML algorithms provide a dynamic and proactive approach to network security, significantly improving the ability of SDN environments to safeguard against a wide range of cyber threats. The integration of BC technology into SDN architectures aims to achieve several key objectives: enhancing operational transparency, fortifying network security, and ensuring control data integrity.

BC technology is a decentralized and distributed digital ledger system characterized by its immutability, transparency, security, smart contracts, tokenization, interoperability, efficiency, anonymity, privacy, and programmability [8,9]. These features make BC a promising technology for various applications in addition to cryptocurrencies (e.g., Bitcoin and Ethereum), including agricultural product traceability [10], healthcare [11,12], renewable energy management [13], education [14,15], Internet of Things (IoT) cybersecurity [16,17,18], and more.

In recent years, BC technology has emerged as a promising solution to security concerns, including those in SDN environments. BC’s decentralized and immutable nature offers a new paradigm for securing network environments [19,20]. By decentralizing the control plane, BC can mitigate the risks associated with SDN’s centralized nature, providing a more resilient infrastructure. BC also introduces transparency and accountability into network operations [21,22], making it more difficult for malicious actors to compromise the system.

However, traditional BC-based applications like Bitcoin [23,24] and Ethereum [25,26] are not without their limitations, particularly when applied to high-speed and high-volume environments like SDN [27,28]. These challenges include scalability, energy consumption, throughput time, and transaction fees [29,30].

While BC technology was originally designed to secure and verify human transactions, another type of DLT, IOTA Tangle, was designed for the IoT ecosystem. IOTA Tangle is optimized for machine-to-machine communication and supporting microtransactions. The emergence of the IOTA Tangle represents a revolutionary shift in DLT. In contrast to traditional BCs, which rely on a linear chain of blocks, the IOTA Tangle employs a directed acyclic graph structure to address the scalability and efficiency issues associated with conventional BCs. The IOTA enables the parallel processing of multiple transactions, eliminating the need for miners and significantly reducing transaction fees and energy consumption [31,32]. The distinct features of the IOTA Tangle include high scalability, feeless transactions, fast transaction speeds, and low energy consumption. These features make it a promising technology for a diverse range of applications, e.g., IoT [33,34], healthcare [35], industrial sectors [36,37], and federated learning [38]. The introduction of IOTA 2.0 and its smart contract capabilities further extends its applicability, making it a suitable candidate for enhancing SDN security.

This paper primarily focuses on enhancing the scalability of Access Control systems in SDN environments through the innovative use of IOTA 2.0 smart contracts. While security and energy efficiency are also critical aspects of our approach, the central objective is to demonstrate how IOTA’s unique architecture can significantly improve the scalability of Access Control mechanisms, enabling SDN environments to efficiently handle larger and more complex networks. The original contributions presented in this research are as follows:

- **Overcoming BC challenges**: By employing IOTA 2.0 Tangle, we overcome the scalability, energy consumption, throughput time, and transaction fee challenges inherent in traditional BC-based solutions for securing SDN environments.
- **Automating SDN management**: We use IOTA smart contracts to automate and secure the management and operation of the SDN network.
- **Strengthening Access Control**: We use an authority smart contract as a CA to define and verify trusted entities, and we implement SC-based Access Control to manage interactions between controllers and switches within the SDN, ensuring secure and authorized communication.
- **Ensuring control data integrity**: Our approach leverages IOTA 2.0’s Tangle architecture, decentralized consensus, and the immutability of transactions to guarantee the integrity of control data in SDN, protecting it from unauthorized modifications, and ensuring reliable operations.

We structure the remainder of this paper as follows: Section 2 gives an overview of recent studies on the use of BC technology to enhance SDN security. Section 3 gives the necessary background, providing an overview of SDN security challenges and IOTA 2.0 smart contracts. Section 4 presents the IOTA 2.0 smart contracts-based system for fortifying the security of SDN. Section 5 focuses on the practical implementation of IOTA 2.0 SCs within SDN environments and presents a comprehensive analysis of the results obtained. Section 6 concludes the paper by summarizing the key findings and contributions of the research.

## 2. Related Work

This section provides a comprehensive review of recent research focusing on the application of distributed ledger technologies (DLTs), e.g., BC, to enhance the security of SDN.

Weng et al. [39] proposed a BC-based monolithic secure mechanism to enhance SDN security by decentralizing the control plane, ensuring the authenticity and accountability of application flows, implementing Access Control mechanisms, and integrating secure protocols with smart contracts on the BC. By recording network events on the BC, the mechanism enables the traceability and auditing of network behaviors, addressing single-point failures and improving scalability in SDN environments. The paper concludes that this innovative approach offers a comprehensive solution to SDN security challenges, leveraging BC technology to provide a secure, decentralized, and accountable framework for network management and control.

Pourvahab and Ekbatanifard [40] presented a novel forensic SDN–IoT architecture that utilizes BC technology to enhance security and efficiency in digital forensics processes within IoT environments. The proposed architecture demonstrates a superior performance in terms of delay, throughput, accuracy, response time, processing time, and security compared to previous works. The study emphasizes the importance of BC in ensuring data integrity by preventing tampering, and establishing a secure chain of custody for digital evidence. Future validation plans include testing the architecture in a large-scale network environment and implementing additional authentication and load-balancing mechanisms.

Yazdinejad et al. [41] presented a novel approach to enhancing security in SDN through the BC-enabled packet parser (BPP) architecture. By leveraging BC technology and FPGA hardware, the BPP architecture demonstrates efficient attack detection capabilities with a low false-positive rate and a high detection rate. The study emphasizes the importance of integrating security measures into both the control and data planes of SDN networks, as well as BPP’s potential to improve network security by detecting and communicating attacks to the SDN controller.

Aujla et al. [42] explored the integration of BC technology with SDN to address the challenges faced by smart cities, such as channel congestion and limited scalability. By proposing BlockSDN as a solution, the study aimed to enhance data transmission efficiency and security in smart city environments. It emphasizes the role of SDN in providing improved bandwidth capabilities and flexibility for dynamic data transmission requirements. Additionally, the paper highlights the security concerns associated with SDN architectures, particularly the vulnerability of the centralized controller to attacks.

Shashidhara et al. [43] introduced SDN-chain, a BC-based privacy-preserving protocol for software-defined networks, aiming to address the vulnerabilities in existing security protocols such as ARP poisoning and DDoS attacks. By integrating BC technology, SDN-chain enhances network reliability, safety, and decentralization, mitigating the risks associated with centralized SDN controllers. The Ethereum BC implements a delegated proof of stake algorithm to support the initialization, registration, and authentication phases of the proposed security model. Through informal security analysis and simulations, SDN-chain demonstrates an improved network efficiency with reduced delay and bandwidth.

Algarni et al. [44] introduced BCNBI, a BC-based security framework for the northbound interface in SDN, aiming to enhance security by addressing the confidentiality, integrity, and availability concerns. BCNBI utilizes a light-weight BC architecture to authenticate applications and the SDN controller, enforce Access Control policies, and monitor the application behavior. Compared with existing solutions and demonstrating its superior performance in handling transactions, BCNBI showcases its efficiency in securing the SDN environment.

Kovacs et al. [7] investigated a range of critical topics concerning network optimization and security within the realm of BC-enabled SDN controllers and IoT deployments. The research delved into secure storage and access for task-scheduling schemes on consortium BC and the Interplanetary File System, as well as the development of proof-of-authentication mechanisms for scalable BC in resource-constrained distributed systems. Furthermore, the paper explored cooperative traffic control schemes among ISPs using bargaining game approaches, analyzed the impact of zero-rating content on the Internet’s quality of service, introduced machine learning-based action recommenders for network operation centers, and discussed enhancements in SDN security for IoT deployments through BC integration.

The aim of this paper is to enhance the security of SDN environments by leveraging IOTA 2.0 smart contracts. Our proposed system introduces three separate smart contracts, namely Authority, Access Control, and DoS Detector, to provide robust security mechanisms that ensure secure network operations, prevent unauthorized access, and mitigate DoS attacks. By utilizing the IOTA Tangle’s directed acyclic graph structure, our approach aims to enhance scalability, efficiency, and energy consumption while eliminating transaction fees. To validate the efficacy of IOTA 2.0 smart contracts in providing a decentralized and efficient solution for securing SDN environments, we conducted comprehensive simulations using Mininet and the ShimmerEVM IOTA test network.

Table 1 presents a comparative analysis of our proposed IOTA 2.0 smart-contract-based system compared to existing systems using DLTs to improve SDN security.

## 3. Background

This section explores the key research areas, focusing on the security challenges in SDN and presenting IOTA 2.0 SCs as an innovative solution to address these issues.

### 3.1. Comprehensive Analysis of SDN Security Challenges

**Figure 1:**
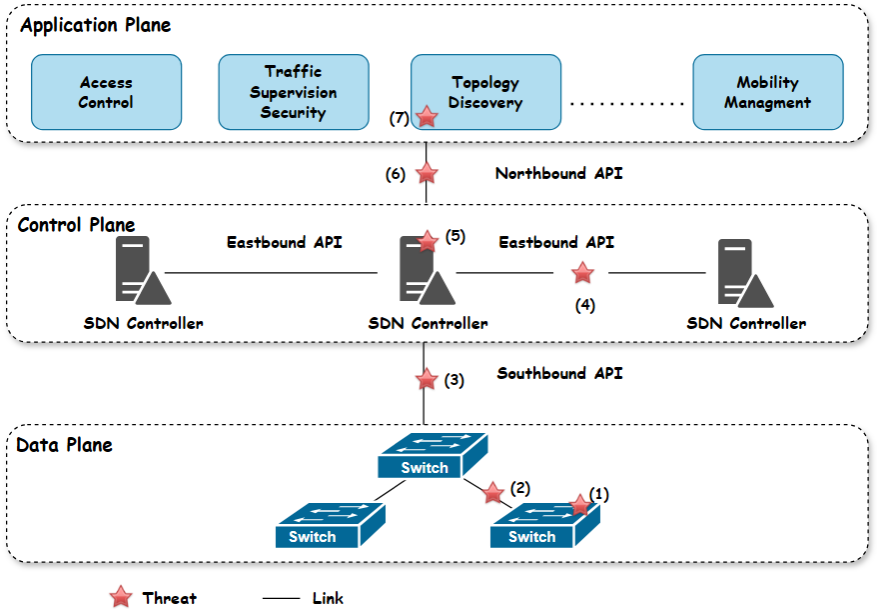
Security threat and vulnerability analysis in SDN by layer.

SDN provides a significant advancement in network management by decoupling the control plane from the data plane, and thus enhancing the programmability and flexibility. However, it also introduces security challenges due to its unique structural design [4,45,46], presenting various threats and vulnerabilities across different network layers and interfaces, as shown in Figure 1.

1. **The SDN switch**, a hardware and software device, is susceptible to threats like flow table modification, topology spoofing, and DDoS attacks, which can insert malicious nodes or modify flow rules.
2. **With regard to the link between switches,** the SDN architecture’s lack of encryption on the links between SDN switches allows hackers to intercept information, thereby compromising network security.

1. **The eastbound interfaces** are vulnerable to security threats due to the lack of encryption on the links connecting controllers. This vulnerability compromises the integrity of inter-controller communications, allowing hackers to manipulate network behavior and share false information.
2. **SDN controllers** face security challenges like DDoS attacks, unauthorized access, and interception risks due to their centralized architecture. The lack of standardized protocols exacerbates these vulnerabilities, allowing attackers to alter network topology and hack switches.
3. **The northbound interface**—a communication link between applications and controllers—is susceptible to security breaches due to weak authentication and inappropriate authorization. This can enable identity theft and unauthorized access, leading to flow modifications and processor overload.
4. **The applications plane** faces security challenges due to its role in managing network behaviors and policies, lack of robust authentication and Access Control mechanisms, direct interaction with SDN controllers, and standardized security protocols.

### 3.2. Overview of IOTA 2.0 Smart Contracts

BC technology offers benefits like decentralization, security, and transparency, but also faces challenges like scalability, high energy consumption, transaction fees, throughput time, and network latency [47,48] in IoT devices. The IOTA Tangle [49], an alternative DLT for the IoT ecosystem, implements a directed acyclic graph structure for parallel transaction processing and scalability.

The IOTA 2.0 Tangle [50] has undergone significant improvements to address scalability, security, and decentralization challenges. Its architecture, consensus mechanisms, and functionality have been redesigned to address the limitations of previous releases [51,52]. Tangle, a novel DAG data structure for IoT, offers immutable data, fee-less microtransactions, low resource consumption, and security based on PoW consensus. IOTA 1.5 (Chrysalis) [53] improves the security and usability of IOTA 1.0 by introducing improvements like better tip selection, autopeering, atomic transactions, adoption of the UTXO model [54], increased throughput, and faster confirmations.

IOTA 2.0 [55] is the first fully decentralized version of the network, incorporating SCs and a decentralized consensus mechanism, and allowing network nodes to independently validate transactions and achieve consensus without a central authority [56].

Coordicide has numerous features, including the following:

- **Tangle technology**: Coordicide employs a Tangle-directed acyclic graph for parallel transaction processing, enhancing scalability and TPS compared to traditional BCs’ linear chain of blocks.
- **Decentralization and scalability**: IOTA 2.0 eliminates the Coordinator, a special node for transaction validation. Moving towards a fully decentralized system enhances the network’s scalability and security.
- **Energy efficiency**: Tangle’s design simplifies the transaction validation, reduces computational power, and makes IOTA more energy-efficient compared to traditional proof-of-work BC systems by eliminating the need for miners.
- **No transaction fees**: IOTA 2.0 maintains its no-fee transaction feature. This feature makes microtransactions viable and opens up a range of applications, particularly in the Internet of Things domain.
- **Interoperability**: IOTA Tangle 2.0 facilitates the transfer of value between different BC networks due to its interoperability with other BC platforms.
- **Smart contract capabilities:** IOTA 2.0 enhances its platform’s competitiveness in DLTS by enabling developers to create complex decentralized applications using SCs.

IOTA 2.0 SCs constitute a decentralized network designed for IoT applications, offering enhanced security, scalability, and suitability. They operate on a distributed network with multiple validators, going through four phases: creation, deployment, execution, and completion. The protocol (ISCP) uses programming languages like Solidity and subchains linked to the main Tangle, reducing the reliance on the main network. This setup supports parallel execution, inter-chain communication, and an Ethereum virtual machine.

**Figure 2:**
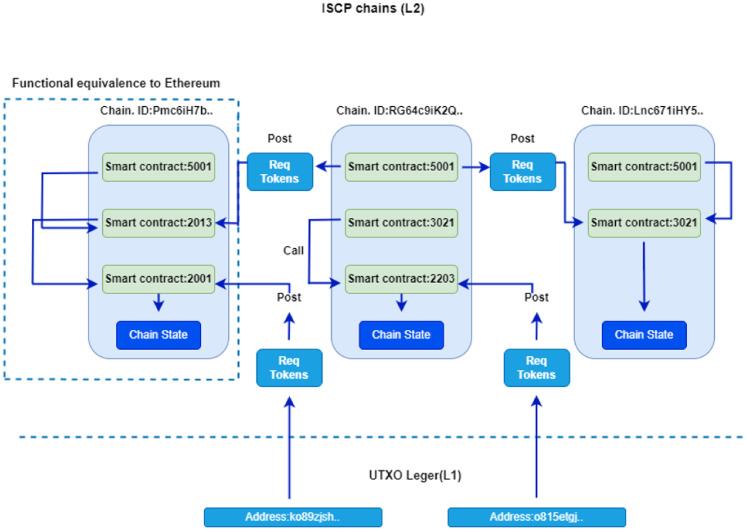
IOTA smart contract protocol chains.

Figure 2 illustrates ISCP chains that manage state and contract execution, with validator nodes validating state changes and publishing them to Layer 1. This setup reduces the transaction costs, minimizes the network strain, and supports Solidity-based contracts. IOTA SCs enhance scalability and support complex contracts, operating on Layer 2 within the IOTA multi-asset ledger.

IOTA Tangle has created new opportunities for various application domains due to its unique smart contracts, feeless transactions, and fully decentralized nature. These domains include healthcare [35,57], Industry 4.0 [37,58], the Internet of Things (IoT) [59], and autonomous IoT systems [60].

Table 2 concludes the subsection with a comprehensive comparative study between IOTA 2.0 and well-known BC-based cryptocurrencies, specifically Bitcoin [61], Ethereum [62,63], and Hyperledger [63]. Table 2 provides a comparative study of IOTA 2.0 and other BC-based cryptocurrencies.

## 4. Proposed IOTA–SDN System

**Figure 3:**
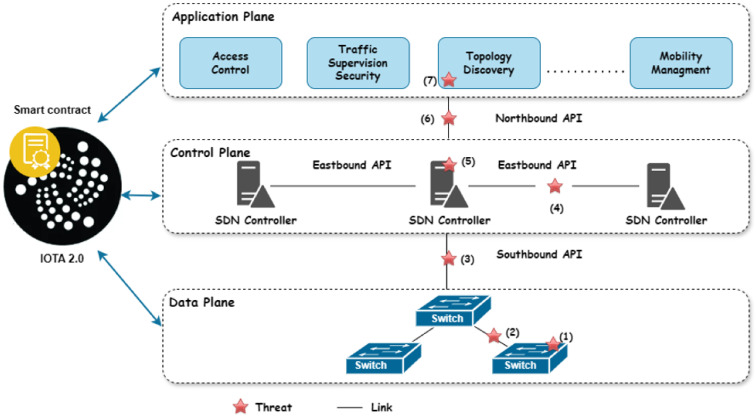
IOTA-based SDN.

This section presents our innovative proposal for an IOTA-based system designed to effectively manage and secure SDN. As illustrated in Figure 3, the architecture of our IOTA-based SDN, where SDN controllers play a central role, guarantees secure horizontal and vertical communication with switches.

IOTA 2.0 has incorporated resilient mechanisms to mitigate the consequences of denial-of-service (DoS) attacks. Nevertheless, ongoing development and testing indicate that the network is not completely immune to such threats. The IOTA Foundation and its community are continually striving to enhance the network’s security and scalability, finite resources that diminish over time, and thus impeding their prolonged accumulation. Furthermore, we proposed integrating a smart-contract-based DoS Detector, which is critical in proactively countering potential threats, thereby strengthening the system’s security posture.

**Figure 4:**
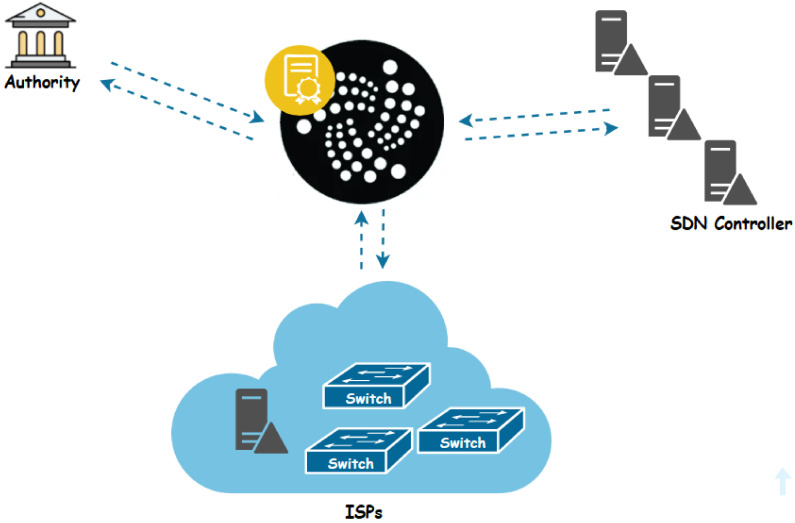
IOTA-based system for SDN.

Our system involves ISPs, with each overseeing its own dedicated controller linked to a set of switches, functioning as primary administrators and standby controllers for other domains. Collaborative efforts among ISPs are essential to extend network coverage across various ISP domains. It is imperative to monitor this collaboration to prevent any ISPs from violating regulations or operating independently within the network. The contracts we designed establish an Access Control framework for controllers, ensuring secure, regulated, and well-organized collaboration in the network’s operations. This ecosystem involves three key actors, as illustrated in Figure 4.

- **The Authority:** Functioning as a Certificate Authority (CA), the Authority holds pivotal responsibility in overseeing the involvement of trusted entities, specifically ISPs, within our proposed system. Its primary role lies in ensuring the exclusive authorization of an ISP to integrate its controller, switches, and standby controller components. Moreover, the CA serves as a cornerstone in upholding the security and integrity of the system by meticulously managing the authorization procedures for these entities. Furthermore, it defines the expiration parameters of digital certificates and offers essential revocation services to invalidate non-expired certificates when necessary.
- **ISPs:** The Authority acting as CA approves only trusted entities (ISPs) to access our system. Each ISP assumes a critical role, maintaining its controller and switches. These controllers serve as primary administrators, and are intricately connected to a network of switches, facilitating efficient data transmission and network management. Notably, ISPs wield the Authority to manage access permissions, authorizing or withdrawing access and integrating or excluding backup controllers across different network domains.
- **The SDN controller:** Within the system architecture, the controller assumes a dual role of paramount importance. Firstly, it functions as the primary administrator within its designated domain, overseeing and orchestrating network operations, managing data flow, and ensuring the smooth functioning of connected switches. As the primary administrator, the controller holds authoritative control over the domain’s network infrastructure, making critical decisions to optimize performance and maintain security. Additionally, the controller assumes the crucial responsibility of serving as the standby controller for other domains within the system. In this capacity, it stands ready to assume control in the event of a primary controller failure or disruption [64].

### 4.1. Overview of the Architecture and Components of the Proposed System

**Figure 5:**
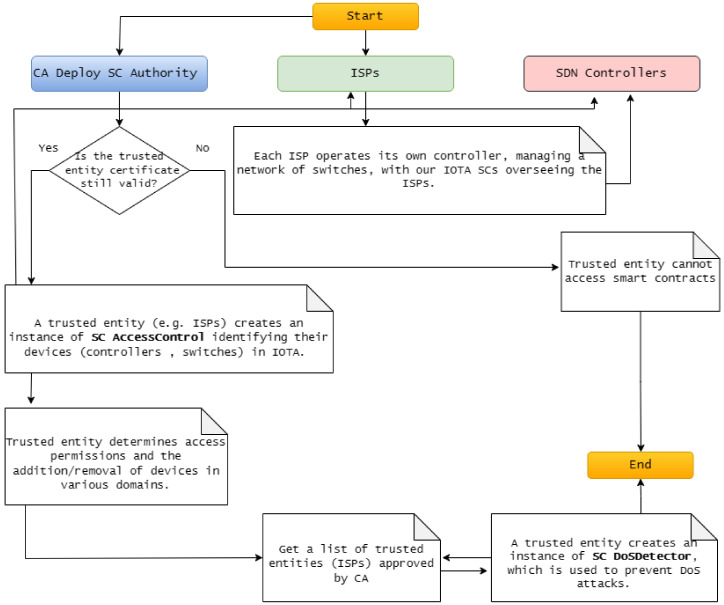
The workflow of our proposed model.

The workflow of our proposed model involves actors and SCs in IOTA-based SDN, which is implemented by three SCs illustrated in Figure 5.

We clarify our solution’s workflow by involving the actors and the previously presented SCs. Initially, the CA deploys an Authority SC instance in IOTA and maps trusted entities (ISPs) by linking their IOTA addresses to their public key certificates. Each ISP then creates an Access Control SC instance to manage its devices (SDN controllers and switches), determine access permissions, and manage devices. Once the CA approves a list of trusted entities, the ISP creates a DoS Detector SC instance to protect against DoS attacks.

Finally, each CA-approved ISP operates its own SDN controller, managing a network of switches. These SDN controllers serve as primary administrators and standby controllers for other system domains. Furthermore, our system facilitates collaboration among ISPs to extend network coverage across various ISP domains, with our SCs overseeing the ISPs to secure SDN environments.

### 4.2. Authority Smart Contract of CA

**Figure 6:**
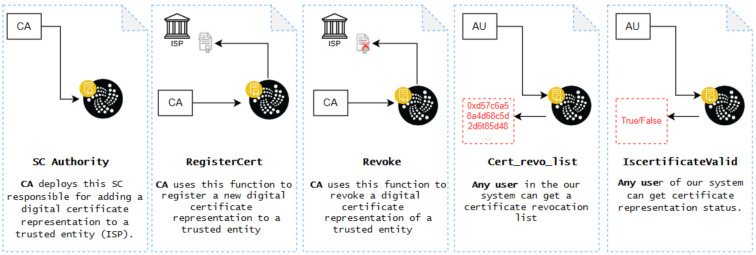
The workflow of the Authority SC along with the actors involved in IOTA.

The Authority smart contract is designed to manage and approve trusted entities, specifically Internet Service Providers (ISPs), that are authorized to participate in our proposed system. To clarify its function, we can provide a practical example where the Authority smart contract maps the public key certificates of ISPs to their IOTA addresses. This mapping ensures that only authorized ISPs can integrate their network components, such as controllers and switches, into the SDN ecosystem. For instance, if an ISP seeks to join the network, the Authority smart contract verifies the ISP’s digital certificate, ensuring that it is valid and issued by a trusted Certificate Authority. Upon approval, the ISP is granted access to the network, where it can deploy and manage its SDN controllers and switches securely. This example demonstrates how the Authority smart contract plays a crucial role in maintaining the security and integrity of the SDN environment by controlling the entry of trusted entities. Figure 6 illustrates the functions within the SC and the actors involved.

Rectangles labeled “CA” in steps 1, 2, and 3 denote Certificate Authority, while those labeled “AU” in steps 4 and 5 represent any user within the IOTA-based SDN. Step 2 provides the registration certification representation for the ISP, while step 3 presents the revoke registration certification representation for the ISP. Step 4 offers a list of revocation certifications, and step 5 presents the certification representation status.

Further details about the SC Authority are presented in the simulation results section.

### 4.3. Access Control Smart Contract of CA

The Access Control smart contract is implemented to manage the permissions and access rights between controllers and switches within the SDN environment. To provide a clearer understanding, we will include a practical example where an ISP uses the Access Control smart contract to oversee and regulate network device interactions. For instance, within an ISP’s domain, the Access Control smart contract could be used to grant a controller permission to communicate with specific switches based on predefined policies. If the controller needs to establish communication with another domain’s controller for load balancing, the Access Control smart contract can dynamically adjust the access permissions to allow or revoke this interaction. This real-world scenario illustrates how the Access Control smart contract ensures that network devices operate within secure boundaries, preventing unauthorized access and maintaining the overall security of the SDN environment.

**Figure 7:**
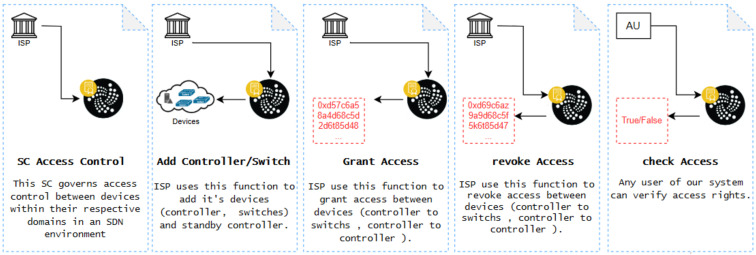
The workflow of the Access Control SC along with the actors involved in IOTA.

Figure 7 shows the functions in the SC, along with the actors involved.

The ISP, approved by CA in steps 1, 2, 3, and 4, is recognized as a trusted entity. Step 2 involves presenting devices such as SDN controllers and switches. Step 3 entails furnishing a list of granted access between devices (controller to switch, controller to controller). Step 4 involves providing a list of revoked access between devices (controller to switch, controller to controller). Step 5, denoted as “AU”, represents any user in the IOTA-based SDN. The simulation results section provides more information about the Access Control SC.

### 4.4. DoS Detector Smart Contract of CA

**Figure 8:**
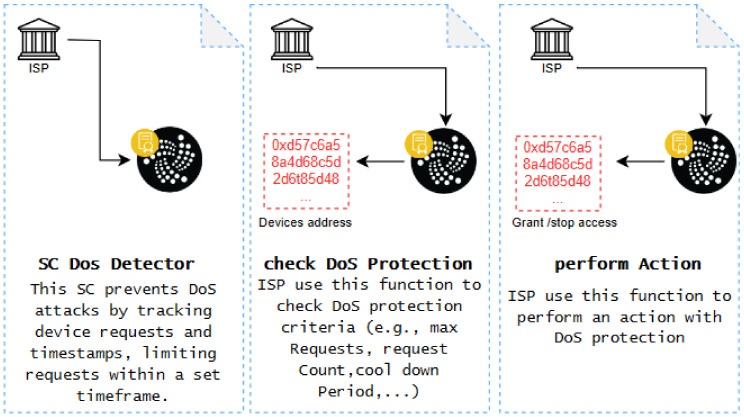
The workflow of the DoS Detector SC along with the actors involved in IOTA.

The DoS Detector smart contract is designed to protect the network from denial-of-service (DoS) attacks by monitoring the frequency and volume of requests made by network devices. To better illustrate its function, we propose adding a case study example where the DoS Detector smart contract is deployed in an SDN environment to monitor request activity. For instance, consider a scenario where a malicious entity attempts to overwhelm the network by sending an excessive number of requests to a controller within a short period. The DoS Detector smart contract tracks these requests, and if the number exceeds a predefined threshold, the contract temporarily blocks further requests from the offending device, effectively mitigating the attack. This practical example demonstrates how the DoS Detector smart contract actively safeguards the network by enforcing rate limits and preventing potential disruptions caused by DoS attacks. Figure 8 shows the functions in the SC along with the actors involved.

The CA establishes the ISP as a trusted entity in steps 1, 2, and 3. Step 2 evaluates the DoS protection criteria, including maximum requests, timestamp, request count, and cooldown period, for each device address. Step 3 entails taking action (granting or stopping access) based on the DoS protection criteria. The simulation results section provides more information about the DoS Detector SC.

### 4.5. Key Benefits of the Proposed System

Our innovative system integrates an IOTA 2.0 layer, enhancing the security of the SDN infrastructure. Utilizing SCs ensures robust security for both horizontal and vertical communication channels. This system also establishes a trusted entity to oversee its domain, including controllers, switches, and standby controllers. This trusted entity meticulously manages access permissions in collaboration with other accredited entities, bolstering the system’s overall security framework. Furthermore, our proposed model includes a CA serving as the trusted service provider for safeguarding the SDN infrastructure. It achieves this by authenticating trusted entities through the mapping of their IOTA addresses to their corresponding public key certificates. Specifically, our proposal entails the inclusion of trusted entities possessing valid certificates authorized by the CA, permitting them to actively engage within our system.

## 5. Simulation Results and Discussion

In this section, we present the simulation results and discuss the effectiveness of our proposed system. The simulation environment was carefully constructed using various platforms and tools to evaluate the integration of IOTA 2.0 smart contracts within an SDN environment. The following subsections provide an overview of the platforms used, the simulation setup, and an analysis of the results obtained from the tests.

### 5.1. Simulation Environment

#### 5.1.1. Platforms Used

**Figure 9:**
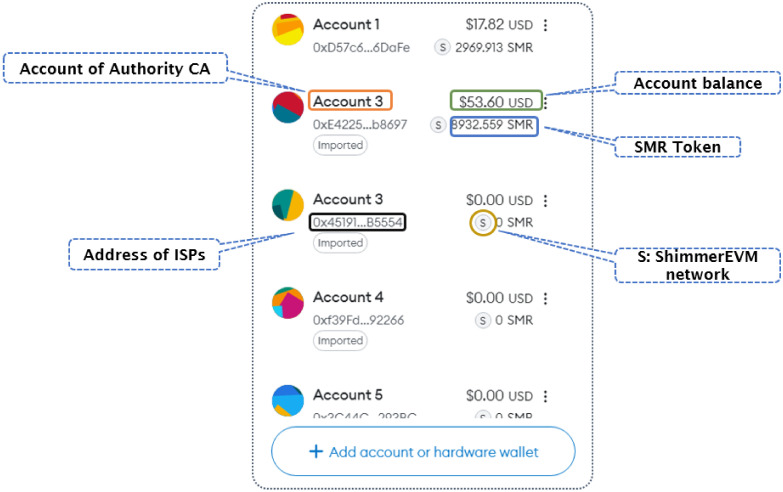
Account balance after adding the ShimmerEVM Network and obtaining SMR funds in the MetaMask wallet.

- **Mininet**: is a leading emulator in the field of SDN, providing academics and developers with a flexible platform for creating virtual networks, exploring SDN concepts, and examining network applications. Mininet effortlessly combines with prominent SDN controllers like OpenDaylight, ONOS, and Ryu, enabling customers to evaluate the effectiveness of their SDN applications across various controller platforms. Mininet (version 2.3.1b4) was selected because it is a widely used SDN emulator that allows for the creation of virtual networks, enabling the testing and development of network applications. It supports integration with major SDN controllers and is highly valued in academia for its flexibility, ease of use, and ability to efficiently replicate real-world network environments. This made Mininet the ideal tool for simulating our SDN environment, providing a robust and reliable platform for testing our proposed system.
- **ShimmerEVM**: is a test network in the Shimmer ecosystem, that is specifically designed to emulate the Ethereum Virtual Machine (EVM) environment on IOTA’s Tangle 2.0. This network allows developers to deploy and test smart contracts in a simulated Ethereum environment, but with the added benefits of IOTA’s unique features. Unlike traditional BC networks, ShimmerEVM leverages the IOTA Tangle, a DAG structure that enables the parallel processing of transactions, leading to higher scalability, lower energy consumption, and feeless transactions. We chose ShimmerEVM for our project to harness these advantages, allowing us to develop and test our IOTA 2.0 smart contracts in a secure, efficient, and scalable environment before moving them to the IOTA mainnet. After connecting to the ShimmerEVM Network and adding SMR funds to the MetaMask wallet, Figure 9 shows the account balance.

#### 5.1.2. Simulation Setup

Algorithm 1 outlines the setup and execution process for integrating IOTA 2.0 smart contracts within an SDN environment using Mininet. The steps involve configuring the necessary tools and networks, deploying smart contracts on the ShimmerEVM Test Network, and validating the integration to enhance the security and efficiency of SDN operations (see Appendix A). This structured approach ensures a seamless interaction between the SDN components and IOTA’s DLT, enabling secure and scalable network management.
**Algorithm 1** Simulation setup for IOTA–SDN integration.1:**Initialize MetaMask and Connect to ShimmerEVM Network**2:Set network parameters:
Network name: ShimmerEVM
RPC URL: https://json-rpc.evm.shimmer.networkChain ID: 148Currency symbol: SMRExplorer URL: https://explorer.evm.shimmer.network3:Fund EVM account using EVM Toolkit and transfer SMR funds to MetaMask account.4:**Deploy smart contracts on ShimmerEVM Network**5:Open Remix IDE, compile solidity smart contracts, and deploy them to ShimmerEVM Network.6:**Configure Mininet for SDN simulation**7:Define network topology:Topology 1: 1 controller, 2 switches, 4 hosts.Topology 2: 1 controller, 2 switches, 4 hosts.8:Initialize network infrastructure by linking controllers, switches, and hosts.9:Start SDN controllers and establish network communication.10:**Integrate IOTA 2.0 with Mininet network**11:Use Python libraries to connect to the Shimmer Network.12:Deploy IOTA 2.0 smart contracts within the Mininet environment.13:Configure smart contracts for seamless operation with network components.14:**Validate integration**15:Ensure secure communication among network devices using IOTA 2.0 smart contracts.16:Monitor network behavior to verify correct deployment and operation of smart contracts.17:**Run simulation and record results**18:Execute various network scenarios using the configured Mininet environment.19:Collect data on network performance, security, and transaction processing times.20:Analyze results to evaluate the effectiveness of IOTA 2.0 in enhancing SDN security.

Table 3 provides a summary of the key parameters and tools used in the simulation, highlighting the configuration of the network, the development environment, and specific settings for smart contract implementation.

Our system was developed using Remix IDE and Solidity and deployed on the ShimmerEVM IOTA Test Network. After funding the MetaMask wallet (version 10.26.0) with SMR and connecting to the ShimmerEVM Network, we used Mininet and Python to create a secure and scalable SDN environment integrated with IOTA 2.0.

**Figure 10:**
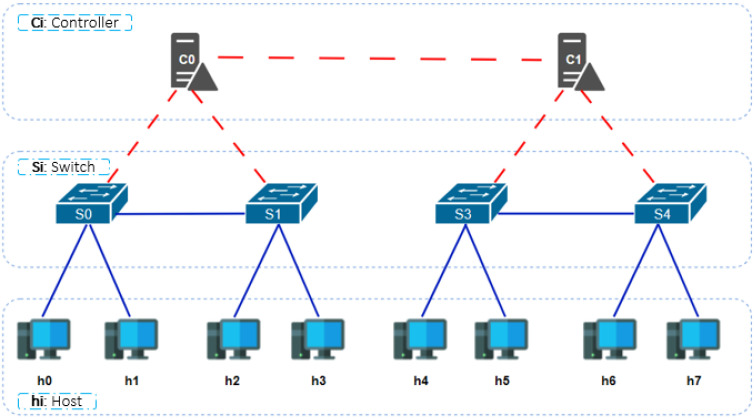
Topology of our system for securing SDN based on IOTA 2.0.

The setup included 14 nodes—2 controllers, 4 switches, and 8 hosts—designed for redundancy, load balancing, and efficient traffic management, as shown in Figure 10. We selected OpenFlow and OVSKernelSwitch for their reliability and compatibility with Mininet, ensuring effective network emulation. Remix IDE and Solidity were chosen for their ease of use and mature ecosystem, facilitating the development and deployment of secure smart contracts.

Our smart contracts controlled the SDN environment by regulating the participation of trusted ISPs, each represented by a certificate issued by our Authority (CA) smart contract. Access Control between controllers and switches was managed within their domains, while the DoS Detector smart contract monitored devices, enforcing limits on identical requests to prevent DoS attacks.

We tested multiple scenarios to validate our system. First, we verified the connection to the Shimmer Network using Web3 and checked the certificate validity through the *isCertificateValid()* function. Access Control was tested across various devices using functions like *check_access_Controller_to_switch()*, *check_access_Switch_to_switch()*, and *check_access_ Controller_to_controller()*. To counter DoS attacks, we set a limit of two identical requests per minute.

The system was validated through repeated simulations, with an average processing time of 8.42 seconds over 10 trials, ensuring its reliability.

### 5.2. Results and Discussion

#### 5.2.1. Authority Smart Contract

**Figure 11:**
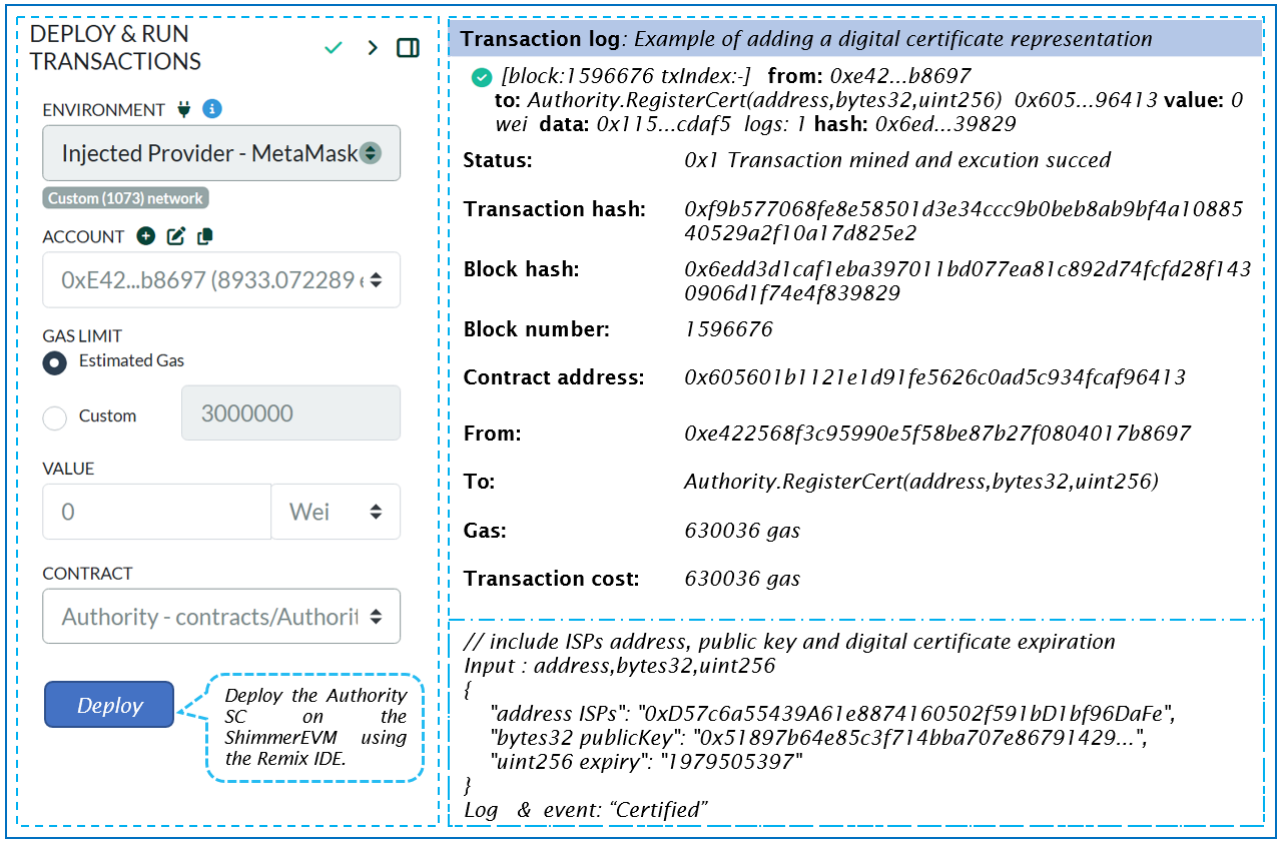
Remix IDE screen of our deployed Authority smart contract.

We use this SC to regulate which trusted entities (ISPs) can participate in our proposed system. Figure 11 details the transaction specifics for deploying an instance of ‘SC-Authority’. This indicates the transaction status, as well as the contract and sender addresses. Additionally, it specifies the transaction destination, which is the smart contract constructor.

**Figure 12:**
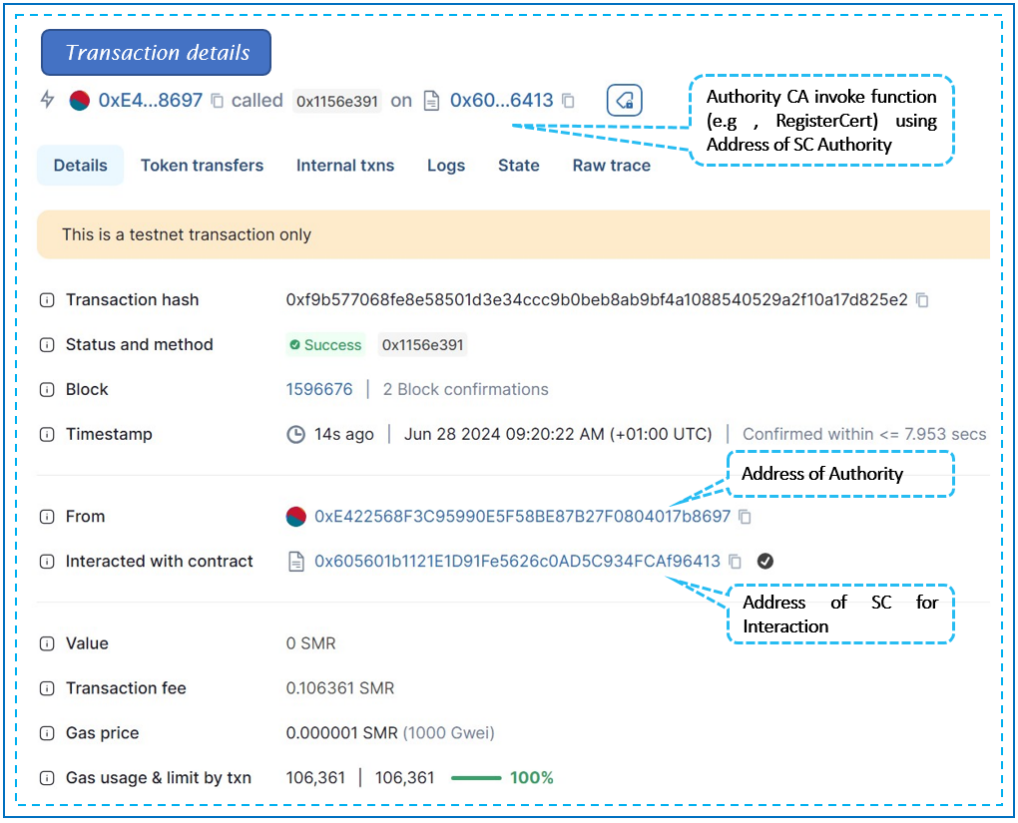
EVM Test Net Shimmer Network screen of interaction with our Authority SC.

Once deployed on the ShimmerEVM Network, the Authority can invoke the SC’s functions using the Authority SC address shown in Figure 11. Specifically, to add a digital certificate representation within the ShimmerEVM Network, the Authority utilizes the *RegisterCert* function. Figure 12 illustrates the ShimmerEVM Network’s deployment and interaction with the Authority SC.

Additionally, events are implemented for each addition in the IOTA network, allowing listening applications to access these events. For example, Listing 1 demonstrates the log of the "certified" event, which is triggered when the smart contract owner executes the ‘RegisterCert’ function.

**Listing 1.** An example of the event register certificate.

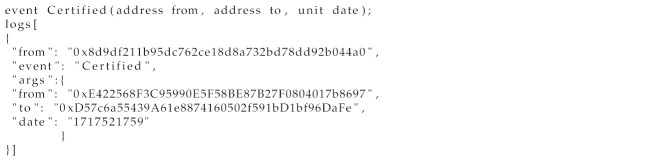



The validation of the ISP certificate’s authenticity is obtained through the *isCertificateValid* function. Additionally, the Authority has the ability to revoke a digital certificate using the *revoke* function. Furthermore, a digital certificate is rendered invalid upon expiration. The function *cert_revo_list* provides an array of addresses belonging to trusted entities whose certificates have been revoked.

#### 5.2.2. Access Control Smart Contract

**Figure 13:**
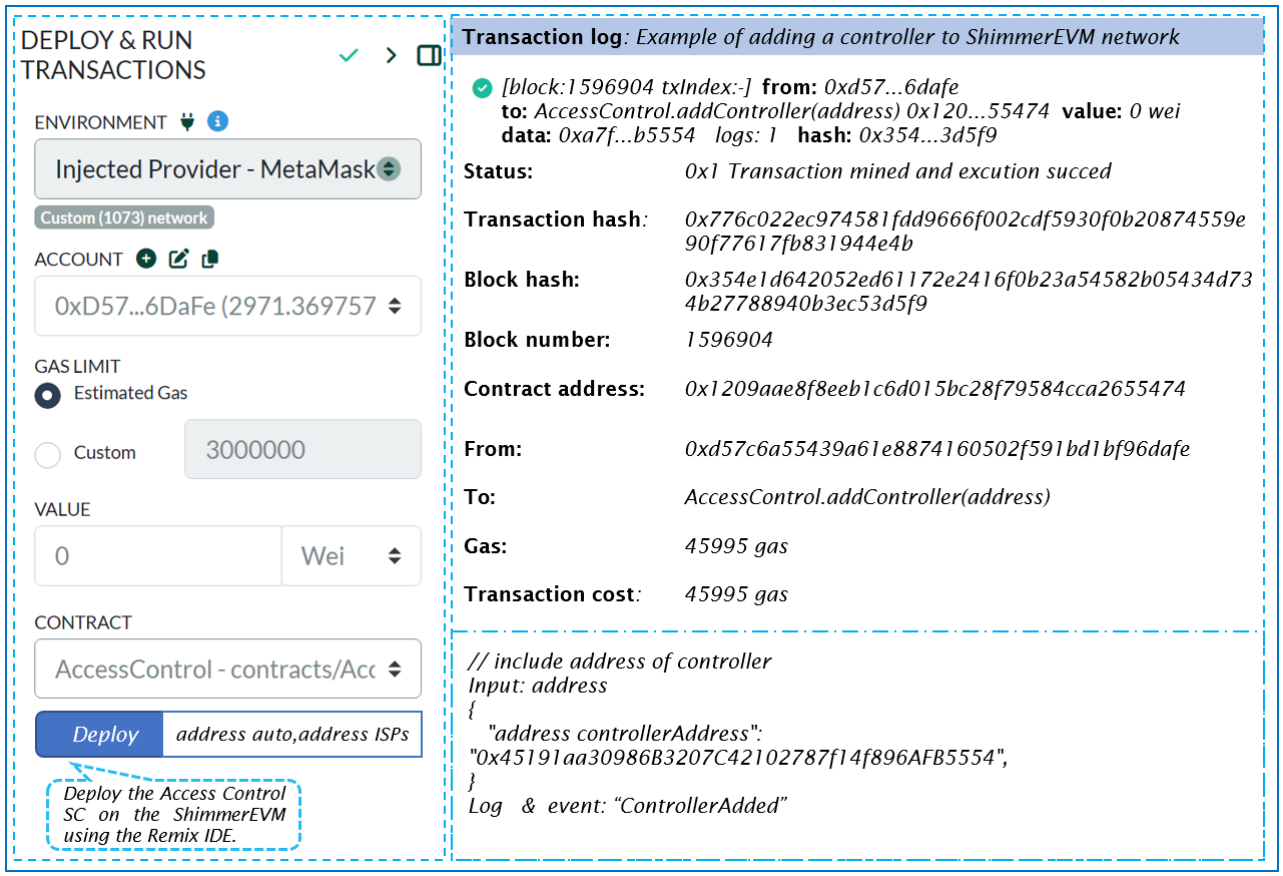
Remix IDE screen of our deployed Access Control SC.

This contract manages Access Control between controllers and switches within their respective domains in an SDN setup. The AccessControl constructor designates ISPs as the owners of the SC, which is achieved by incorporating the address of the previously deployed Authority contract and the address of the trusted entity with a valid certificate. Figure 13 outlines the transaction details for instantiating ‘SC-AccessControl’. It includes the transaction status, followed by the contract and sender addresses, and specifies the transaction’s destination, which is the SC constructor.

Once deployed on ShimmerEVM, ISPs can invoke the SC’s functions using the address of the AccessControl SC shown in Figure 13. Specifically, to add controllers to the ShimmerEVM Network, ISPs utilize the *addController* function.

Listing 2 displays the log of the “ControllerAdded” event that occurs after the SC owner triggers the *addController* function.

**Listing 2.** An example of the event add controller.

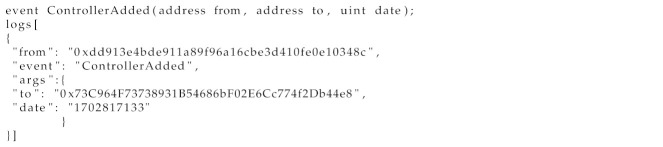



ISP administrators can grant or revoke Access Controls, add or remove standby controllers across domains, and manage controllers and switches within this contract. The contract features functions like *checkAccess* to verify access rights and *grantControllerAccess/revokeControllerAccess* to regulate communication among controllers. These functionalities enhance security and facilitate efficient management within the SDN environment.

#### 5.2.3. DoS Detector Smart Contract

**Figure 14:**
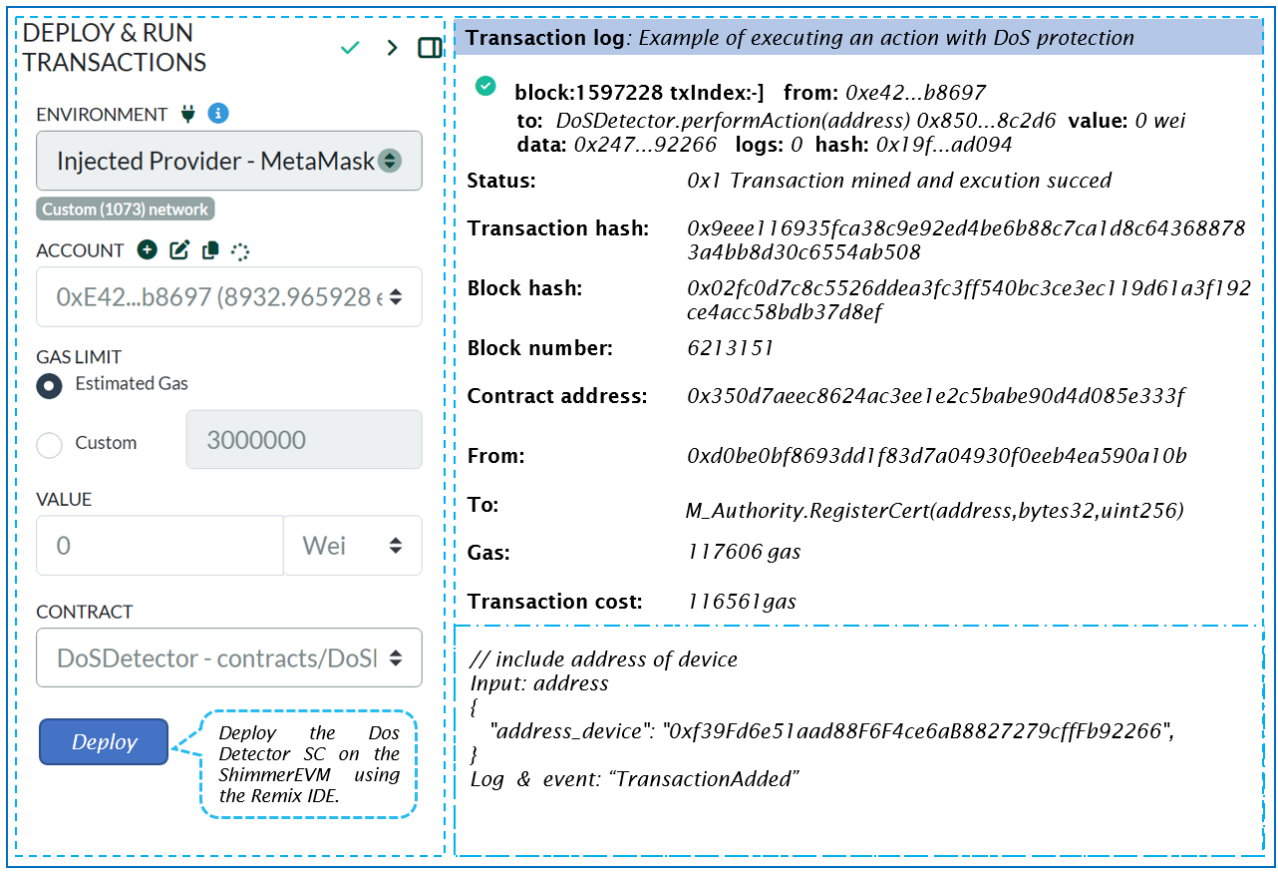
Remix IDE screen of our deployed DoS Detector SC.

This contract monitors individual devices by tracking their request counts and timestamps, enforcing a limit on the maximum number of same requests permitted within a specified cooldown period. Figure 14 outlines the transaction specifics for deploying an instance of the DoS Detector SC. This includes the transaction status, followed by the contract and sender addresses, and details the transaction destination, which points to the SC constructor.

The address of the DoS Detector SC shown in Figure 14 enables access to the contract’s functions once deployed on the ShimmerEVM Network. Specifically, the *performAction* function is used to execute the actions related to DoS protection.

Listing 3 shows the changes made to requests within our system. Specifically, it defines the maximum number of permitted requests within a given time period and sets the cooldown duration in seconds.

**Listing 3.** The adjustment of requests within our system.

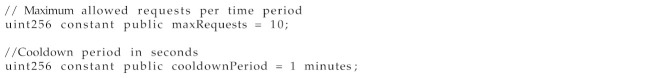



Our SC adopts a comprehensive approach to prevent DoS attacks, which includes checks on frequency, time, Boolean values, and request volumes. Upon calling *performAction*, the contract verifies whether the user’s request count breaches the predefined maximum limit within the cooldown period. If the limit is not exceeded, the action is executed, and both the request count and timestamp are duly updated.

#### 5.2.4. In-Depth Analysis of Results

The simulation results indicate that the integration of IOTA 2.0 smart contracts significantly enhances the performance and security of SDN environments. A detailed examination of the latency and throughput metrics reveals a considerable reduction in transaction processing times, which is largely attributed to the unique architecture of the IOTA Tangle. Unlike traditional BC systems that process transactions sequentially, the DAG structure of IOTA allows for the concurrent validation of multiple transactions.

#### 5.2.5. Potential Explanations for Observed Behaviors

The observed improvements in system performance can be explained by the distinctive features of the IOTA Tangle. The absence of miners and transaction fees reduces the computational overhead and energy consumption, leading to a more efficient network. Additionally, the decentralized nature of the IOTA Tangle minimizes the risks associated with single points of failure, further enhancing the security and reliability of the SDN environment.

#### 5.2.6. Comparative Analysis with Existing Solutions

When compared to traditional BC-based solutions, such as those utilizing Ethereum or Bitcoin [7,39,40,41,42,43,44], the IOTA-based system offers distinct advantages in terms of scalability, energy efficiency, and transaction speed. Traditional BCs often face limitations due to their linear block validation process, which can lead to increased latency and higher energy consumption (see Table 4). In contrast, the IOTA Tangle’s parallel processing and feeless transaction model provide a more sustainable and scalable solution for securing SDN environments.

#### 5.2.7. Limitations of the Proposed System

While promising, the proposed system leveraging IOTA 2.0 smart contracts for securing SDN environments presents several challenges. The complexity of the IOTA Tangle could impact the network performance and scalability, particularly in large-scale deployments, potentially increasing latency and affecting quality of service. The security of the system heavily depends on the proper implementation of smart contracts, which, if flawed, could introduce vulnerabilities. Integration with existing SDN infrastructures may require significant modifications, posing challenges in terms of resource allocation and complexity. Additionally, the system’s reliance on IOTA Tangle may involve a steep learning curve for developers and administrators, with potential issues in computational and energy efficiency. Finally, the decentralized nature of IOTA raises concerns about compliance with data privacy regulations, which could complicate its adoption in regulated environments.

#### 5.2.8. Ethical and Security Considerations

In deploying the proposed IOTA 2.0 smart-contract-based system in real-world SDN environments, it is essential to address the ethical and security implications associated with its use. One of the primary ethical concerns is the potential for the misuse of the system, particularly in environments where privacy and data integrity are paramount. To mitigate these risks, it is recommended that strict Access Controls and encryption protocols be implemented, ensuring that only authorized entities can interact with the network. Additionally, the system must comply with relevant data protection regulations, such as GDPR, to safeguard personal information and maintain user trust. It is crucial to regularly update and audit the smart contracts to identify and address any vulnerabilities that could be exploited by malicious actors. Furthermore, deploying this technology in a way that maintains transparency and accountability is vital for ensuring that it does not inadvertently reinforce existing power imbalances or lead to unintended consequences in the broader network infrastructure. By adopting these recommendations, the system can be deployed ethically and securely, contributing positively to the advancement of SDN technologies.

## 6. Conclusions and Future Work

This research presents an innovative approach to securing SDN environments using IOTA 2.0 smart contracts. By leveraging the IOTA Tangle, our proposed system enhances scalability and efficiency while eliminating transaction fees and reducing energy consumption. We introduced three smart contracts—Authority, Access Control, and DoS Detector—to ensure secure network operations, prevent unauthorized access, and mitigate denial-of-service attacks. Comprehensive simulations using Mininet and the ShimmerEVM IOTA Test Network demonstrated the efficacy of our approach in enhancing SDN security. Our findings highlight the potential of IOTA 2.0 smart contracts to provide a robust, decentralized solution for securing SDN environments. Our approach offers a scalable and efficient solution, addressing the limitations of traditional BC-based systems. However, to further enhance the robustness of our system, future work will focus on integrating machine learning (ML) and deep learning (DL) techniques to develop adaptive and intelligent security mechanisms. Specifically, we plan to design ML/DL models that can dynamically identify and mitigate evolving cyber threats, such as distributed denial-of-service (DDoS) attacks, by learning from network traffic patterns in real time. Additionally, we aim to explore the deployment of reinforcement learning algorithms to optimize the performance of smart contracts under varying network conditions, reducing latency and enhancing decision-making processes.

## Figures and Tables

**Table 1:** Comparison of the proposed system with other existing systems based on DLTs.

Ref.	Focus Area	Key Contributions	DLT	SC	Limitations
[39]	BC-based monolithic secure mechanism for SDN.	Decentralizing control planes, ensuring authenticity and accountability of application flows, Access Control mechanisms, and integrating secure protocols with SCs.	HLF V 1.0	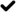	Potential scalability challenges, performance overhead, SC complexity, and interoperability issues. The type of SCs used is not specified.
[40]	Forensic SDN–IoT architecture with BC.	Enhancing security and efficiency in digital forensics, ensuring data integrity, preventing tampering, and securing the chain of custody for digital evidence.	BC	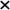	Potential scalability challenges and overhead of BC integration in large-scale SDN environments.
[41]	BC-enabled packet parser architecture.	Enhancing security in SDN through FPGA hardware, efficient attack detection, a low false positive rate, and a high detection rate.	BC	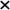	Scalability challenges inherent in BC implementation at the data plane level of SDN networks.
[42]	Integration of BC with SDN for smart cities.	Addressing challenges in smart cities, enhancing data transmission efficiency and security, and improving bandwidth capabilities and flexibility.	BC	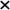	Complexity and potential overhead introduced by integrating BC technology into SDN infrastructures.
[43]	BC-based privacy-preserving protocol for SDN.	Addressing ARP poisoning and DDoS attacks, enhancing network reliability, safety, and decentralization, and reducing delay and bandwidth.	Ethereum	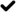	Potential scalability and performance challenges for real-world network operations.
[44]	BC-based security framework for northbound interface in SDN.	Enhancing security by addressing confidentiality, integrity, and availability, authenticating applications and SDN controllers, and enforcing Access Control policies.	BC	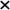	Potential challenges related to scalability, performance overhead, and the computational resources required for BC operations.
[7]	Network optimization and security in BC-enabled SDN and IoT.	Secure storage and access for task scheduling, the development of proof-of-authentication mechanisms, cooperative traffic control, and ML-based action recommenders.	Ethereum	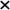	Challenge of scalability and performance issues for large-scale infrastructure networks.
Our system	IOTA 2.0 SCs for securing SDN.	Introducing a novel approach to secure SDN environments using IOTA Tangle and leveraging smart contracts for Authority, Access Control, and DoS Detection.	IOTA 2.0	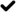	Potential reduction in quality of service, increased latency, and impact on data traffic due to the integration of the DoS Detector smart contract.

**Table 2:** Comparative study between IOTA 2.0 and various BC platforms with SCs and parallel transactions.

Feature/Criterion	IOTA 2.0	Bitcoin	Ethereum	Hyperledger	SEI	Monad	Solana
**Transaction Speed**	Up to 1000 TPS	3–7 TPS	15–30 TPS	1000–10,000 TPS (varies by implementation)	20,000+ TPS	High TPS (specifics TBD)	65,000 TPS
**Scalability**	High	Low	Low	Low	High	High	High
**Energy Consumption**	Very low	High	Medium-high	Low to medium	Low	Low	Low
**Consensus Mechanism**	FPC binary voting protocol	PoW	PoW, transitioning to PoS	PBFT variants, Raft, etc.	Tendermint BFT	Proof of Stake	Proof of History + PoS
**Security Protocols**	EdDSA	ECDSA	ECDSA	ECDSA	EdDSA	EdDSA	EdDSA
**Decentralization**	Fully decentralized	Fully decentralized	Fully decentralized	Permissioned (partially decentralized)	Fully decentralized	Fully decentralized	Fully decentralized
**SC support**	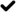	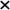	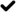	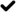	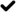	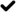	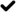
**SC speed**	Fast execution (parallel transactions)	-	Slower execution	Slower execution	Fast execution (parallel transactions)	Fast execution (parallel transactions)	Fast execution (parallel transactions)
**Microtransactions**	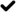	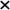	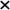	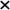	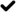	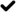	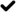
**Transactions fees**	Very low	-	High	High	Very low	Very low	Very low
**Limitations**	Early stage of development Potential network stability issues	Scalability issues, high energy consumption	Scalability issues, gas fees	Limited decentralization, complexity	Early stage of adoption	Early stage of adoption	Complexity, potential centralization concerns

**Table 3:** Simulation details.

Attribute	Value
Simulation time	8.42 s
Number of nodes	14
Network	The ShimmerEVM IOTA Test Network
Mininet controller	OpenFlow
Mininet switch	OVSKernelSwitch
Integrated development environment (IDE)	Remix IDE (version 0.22.2)
Smart contract programming language	Solidity (version 0.8.26)
Interacting with our system	Python (version 3.8)
DoS Detector smart contract simulation **Settings**: max_same_Requests = 2; Cooldown period = 60 s	The device was stopped after 2 transactions during the cooling off period

**Table 4:** Comparative analysis of the proposed IOTA-based system with other existing solutions.

Criteria	Our System (IOTA 2.0)	[39]	[40]	[41]	[42]	[43]	[44]
**Scalability**	High	Medium	Medium	Medium	Medium	Medium	Medium
**Efficiency**	High	Medium	Medium	High	Medium	High	Medium
**Energy efficiency**	Very high	Low	Medium	Medium	Medium	Medium	Medium
**Transaction time**	Fast	Medium	Medium	Medium	Medium	Medium	Medium
**Latency**	Low	Medium	Medium	Low	Medium	Medium	Medium
**Security**	High	High	High	High	High	High	High
**Cost (fees)**	No Fees	Medium	Medium	Medium	Medium	Medium	Medium
**Complexity**	Medium	High	Medium	Medium	High	High	Medium
**Interoperability**	High	Medium	Low	Medium	Medium	Low	Medium

## Data Availability

Dataset available upon request from the authors.

## Abbreviations

The following abbreviations are used in this manuscript:

BC	Blockchain
CA	Certificate Authority
DAG	Directed Acyclic Graph
DL	Deep Learning
DLT	Distributed Ledger Technology
DoS	Denial of Service
ECDSA	Elliptic Curve Digital Signature Algorithm
EdDSA	Edwards-Curve Digital Signature Algorithm
EVM	Ethereum Virtual Machine
HLF	Hyperledger Fabric
IDE	Integrated Development Environment
ISP	Internet Service Provider
IoT	Internet of Things
IOTA	Internet of Things Application
ISCP	IOTA Smart Contracts Protocol
ML	Machine Learning
PoW	Proof of Work
SCs	Smart Contracts
SDN	Software-Defined Networking
TPS	Transaction per Second

## Appendix A

- We presented the complete implementation of our proposed system, IOTA 2.0-based SDN Smart Contracts, at https://github.com/MedFartitchou/SDN_IOTA (accessed on 20 June 2024).
- We tested IOTA 2.0-based SDN Smart Contracts at https://rb.gy/g0esua (accessed on 20 June 2024).
